# Brazilian Food Handlers' Years of Work in the Foodservice and Excess Weight: A Nationwide Cross-Sectional Study

**DOI:** 10.3389/fpubh.2022.869684

**Published:** 2022-05-30

**Authors:** Ingrid C. Fideles, Rita de Cássia C. de A. Akutsu, Priscila R. de F. Costa, Jamacy C. Souza, Rosemary da R. F. Barroso, Raquel B. A. Botelho, Heesup Han, António Raposo, Antonio Ariza-Montes, Alejandro Vega-Muñoz, Renata P. Zandonadi

**Affiliations:** ^1^Department of Food Science, School of Nutrition, Federal University of Bahia, Salvador, Brazil; ^2^Department of Nutrition, Faculty of Health Sciences, University of Brazilia, Brazilia, Brazil; ^3^Department of Science Nutrition, School of Nutrition, Federal University of Bahia, Salvador, Brazil; ^4^College of Hospitality and Tourism Management, Sejong University, Seoul, South Korea; ^5^CBIOS (Research Center for Biosciences and Health Technologies), Universidade Lusófona de Humanidades e Tecnologias, Lisboa, Portugal; ^6^Social Matters Research Group, Universidad Loyola Andalucía, C/Escritor Castilla Aguayo, Córdoba, Spain; ^7^Public Policy Observatory, Universidad Autónoma de Chile, Santiago, Chile

**Keywords:** years of work, foodservice, excess weight, food handlers, Brazil

## Abstract

This study aimed to evaluate the association between the years of work of food handlers in the foodservice and excess weight among Brazilian low-income food handlers. A total of 559 food handlers from all Brazilian regions were characterized using a questionnaire. Weight and height were measured to estimate the Body Mass Index and classify the individuals. The association between food handlers' years of work in the foodservice, anthropometric status, and other variables (gender, age group, educational level, participation in a government program and per capita income at home and energetic consumption) were performed using Pearson's chi-square test (*p* < 0.05). Multinomial logistic regression analyses were performed (*p* < 0.05) as well as sensitivity tests using the outcome continuously and transformed, excluding underweight individuals, in a multivariate linear regression model. Most of the sample was female (63.1%), aged between 21 and 40 years old (63.5%), and 53.3% had studied up to complete elementary school. Almost 41% of the food handlers had less than half the minimum wage per capita income. Of the evaluated individuals, 59.9% presented excess weight. There was an association with family per capita income (Odds Ratio - OR: 1.73; Confidence interval - CI95%: 1.09–2.75); handlers whose per capita income was ≤0.5 minimum wage had a 73% higher chance of obesity than those with higher income. Working in foodservive ≥3 years increased the chance of being overweight by 96% compared to those who work for <3 years (OR: 1.96; CI95%: 1.11–3.49). No significant association was found between the years of work of food handlers in the foodservice and obesity. Since work-related factors may contribute to the high prevalence of excess weight, including working in a food handling environment, the government and employers should consider workplace interventions. These would guide the food handlers in avoiding high rates of excess weight and their consequences on public health. Excess weight is an important driver of costs in the workplace associated with absenteeism, job change, and diseases. More studies are necessary to clarify the relationship between the factors related to work and the anthropometric status of food handlers since excess weight is multifactorial.

## Introduction

Overweight and obesity are characterized by abnormal accumulation of body fat that can harm health. Excess weight is considered an important public health problem worldwide, affecting almost 39% of the global population (about 2 billion people); 0.1 million are obese (1, 2). This scenario contributes to the annual death of about 4 million people worldwide. An imbalance between the individual's food intake and calories spent is one of the leading causes of this condition (1, 2). Following this trend, in Brazil, overweight and obesity have increased over the years. The Surveillance of Risk and Protection Factors for Chronic Diseases Survey (VIGITEL) identified an increase of 12.8% in overweight and 8.5% in obesity from 2006 to 2019 (3), showing that more than half of the Brazilian population is currently in excess weight (55.4%) (3).

Studies suggest a high prevalence of overweight among the different types of workers. In the United States, data indicate excess weight prevalence above 60% among workers (4, 5) and almost 28% of obesity (6). Brazilian studies with factory workers showed excess weight prevalence higher than 50% (7, 8). Besides health problems, gender, socioeconomic status, and income, studies suggest that excess weight prevalence might be higher among food handlers due to easy access to food during the meals' production, favoring a higher caloric intake (9–14). From this premise, it is understood that the more time exposed to this environment, measured by the years of work of food handlers in the foodservice, the greater the possibility of weight gain. A study showed almost 70% excess weight among food handlers in Colombia, concluding that being a food handler was a significant risk factor for excess weight in the evaluated group (12).

In Brazil, about 250,000 workers are employed in food services as food handlers (15). Brazilian studies indicate high overweight among food handlers with prevalences above 50% (9, 11). They present socioeconomic conditions associated with excess weight (low levels of education and income) influencing their food consumption, given the availability and easy access to food high in fat, sugar, and calorie but low in minerals and fiber (9–11). In addition, free access to food due to the activity can favor “pinch” between meal times, increasing food consumption and leading to weight gain (16). Weight gain and low nutrient intake are important risk factors for non-communicable diseases (NCD) development and a higher risk of morbidity and mortality (1). Studies show that excess weight favors the occurrence of chronic inflammation, insulin resistance, arterial hypertension, and dyslipidemia, increasing the risk of cardiovascular events (17, 18). Also, it seems to be associated with musculoskeletal problems (19), and a systematic review revealed high costs due to loss of productivity among workers with excess weight, highlighting absenteeism contributing to the high indirect costs in the short and long terms, with consequences for the employee and the employer (20).

Although studies on adverse outcomes associated with excess weight among workers are relatively frequent, they have limited geographical coverage and few address food handlers. Also, few studies seek to investigate the relationship between the years of work as food handlers in the foodservice area and excess weight. Considering that these professionals work with meals' production, in direct contact with food during their workday, knowing the anthropometric profile of this category and its relationship with the years of work of food handlers in the foodservice can contribute to specific actions that seek prevention and control of their excess weight. Thus, this study aimed to identify the association between the years of work of food handlers in the foodservice and the excess weight of food handlers who work in Brazilian community restaurants by a nationwide cross-sectional study.

## Methods

### Study Design and Participants

This nationwide cross-sectional study was performed with food handlers from Brazilian community restaurants (CR) that provide low-cost meals to the low-income population (21). They offer lunch meals from Monday to Friday in the five Brazilian regions, and employees present the same type of work inside each restaurant. CRs from the Department for Social Development and Hunger Fighting list were eligible for the sample calculation (22).

As inclusion criteria, the restaurant should be part of the CR program. The responsible should sign the Institutional Acknowledgment Agreement. The CR needed to offer at least 500 daily meals. More offered meals indicate more employed food handlers. Therefore, it is important to reach more food handlers in each CR. A sampling plan was calculated from the 65 CRs in the list with a significance level (α) of 5% (23). Using the SAS 9.1.3 program, a minimum of 31 would be selected for the research. Therefore, a random selection was performed in each Brazilian region (North, Northeast, Midwest, South, and Southeast) proportionally to each region's total number of CRs. In total, 36 CRS were visited and included in the study. A minimum of 335 individuals was necessary for a representative sample of CR foods handlers (95% confidence; error of 5%), considering a heterogeneous sample from all 65 CR (2,560 food handlers). In each CR, all the food handlers were invited to participate (*n* = 1,062). Not all the food handlers decided to participate because they did not want to stop their work or were worried about their responses and the maintenance of their jobs. Since the participation is voluntary, we included all food handlers able to participate and who agreed to participate. A total of 559 (52.64%) food handlers participated. The sample has the power of 96.8% to identify the prevalence of overweight, considering 60.8% of excess weight prevalence in the study population as a basis (11, 24).

### Data Collection

After signing the consent form, participants answered a questionnaire with sociodemographic characteristics (gender, age, educational level, per capita income, marital status, smoking, participation in a government program, and years of work as food handlers in the foodservice). Participants were invited to a reserved room before lunch for measurements and asked to take their shoes off and jackets for weight and height. Plenna^®^ (São Paulo, Brazil) weighing scale (150 kg) and stadiometer (220 cm) were used. Body Mass Index (BMI) was calculated.

### Dependent Variable

The study outcome variable (BMI) was categorized into three levels, with low weight and normal weight grouped into one category (due to the low prevalence of the first group-1.43%) (BMI < 25 kg/m^2^) [0: protection], overweight (BMI: ≥25.0 kg / m^2^ < 30.0) [1: first risk category] and obesity (BMI ≥ 30.0 kg/m^2^) [2: second risk category]. Anthropometric indicators commonly used in national and international surveys are based on the weight and height of different age groups. In the case of the adult population, which constitutes the workers, the Body Mass Index (BMI) is the most used indicator. It is recommended by the World Health Organization, which suggests its application for nutritional and population health assessment, given that it is a good indicator for the accumulation of fatty tissue due to excess energy. It presents the same value for both genders and ages (25). Additionally, categorized values allow comparison with other population surveys and studies that use BMI as an indicator of nutritional status from an anthropometric point of view.

### Independent Variables

According to Cáceres-Jerez et al. (12), food handlers presented differences in BMI when carrying out this occupation. A higher BMI was found in those working as food handlers for 36 months or more than those who had less time in this work. Therefore, the main exposure variable was the time that the food handler reported working in the CR, with a cut-off point <36 months [0: protection] and > = 36 months [1: risk] (12).

The analyzed covariables were related to socioeconomic and demographic aspects and included: gender ([0] female and [1] male), age group ([0] < = 40 years and [1]> 40 years), educational level ([0] high school /higher education and [1] Incomplete/complete elementary education), participation in a government program ([0] does not participate and [1] participates) and per capita income at home. For the per capita income, the food handlers were asked about the number of individuals living at home, how many received a salary, and whether the family income was less than, equal to, or greater than a minimum wage. Based on this information, the reported value for family income was divided by the number of household residents. After the calculation, the data were initially grouped into five categories based on the minimum wage (MW) value at the time (R$ 510.00~175.90 USD per month): ≤ ¼ MW (127.50); ¼ to ≤ ½ of the MW (127.51–255.00); >½ to 1 MW (255.01–510.00); 1 to 2 MW (510.01–1020.00); > 2 MW (1020.01). For this article, per capita income was dichotomized into [0]> 1/2 MW (R$ 255.00) and [1] <1/2 MW (R $ 255.00), the cut-off that follows the parameter used in the single registry of families benefited by the public programs in Brazil (26).

The aspects related to health and lifestyle-the presence of NCDs or other diseases ([1] Yes: Diabetes mellitus, hypertension, and others (cancer, dyslipidemia, cardiovascular diseases, respiratory diseases, depression); [0] Absence of NCD); alcohol consumption ([0] no and [1] yes); and tobacco use ([0] no and [1] yes) were self-reported. The variables geographic region of the CR ([0] midwest/south/southeast and [1] North/Northeast) were included. Also, food handlers answered if they received nutritional education in the CR ([0] yes, and [1] no).

### Statistical Analysis

Frequencies and percentages were calculated to describe the categorical variables according to the categories of the outcome variable (BMI). Pearson's chi-square test (χ^2^) was used to identify associations (*p* < 0.05) between covariates to observe a possible indication of collinearity. For all analyzes, a significance level of 5% was adopted.

Given the nominal outcome with three BMI categories, multinomial or polytomous logistic regression was used, a direct extension of the binomial logistic model (19, 27, 28) that applies to outcomes with three or more levels (29). In this type of modeling, the outcome categories have no natural order, and one becomes the reference category. The multinomial logistic regression adjusts simultaneous submodels that compare the other categories of the outcome variable with the chosen reference category. Thus, different logistic regression coefficients are estimated for each independent variable in each outcome variable category. For the present study, the category “low weight/normal weight” was chosen as a reference.

Initially, bivariate analyzes (BMI as a dependent variable) were performed using multinomial regression to examine the variables to be included in the multivariate model (the ones with a *p* < 0.20). The variables smoking and education were associated with outcome (χ^2^) and for examining the effect modification. The model with the primary association (working time and nutritional status), the possible effect-modifying variables (smoking and education), and respective product terms were evaluated for global interaction using the Likelihood Ratio Test, and there was not a global interaction (*p* > 0.05). The goodness of the fit of the model was tested by comparing the complete and incomplete models—without the effect modifier (smoking and education) and the corresponding product terms. The term that proved to be statistically significant and that best adjusted to the primary model, as well as the variables involved were kept in the multivariate model. The interaction concept is centered on the idea that the effect of exposure, compared to a reference category without exposure, may depend on the presence of one or more other exposures (30). Only the term interaction between education and smoking was statistically significant at the end of the analysis, maintained in the adjusted model. The confounding of covariables in the main association was tested (variation in estimates >10%), with gender and NCDs' presence maintained in the final model. The LRT tested the model's goodness of fit, and the final model considered the significance level of the covariates (maintained in the model).

One way to deal with possible violations of assumptions is to conduct a sensitivity analysis. In this sense, the statistical analysis is repeated, using different assumptions to see how sensitive the statistics are to changes in assumptions. It is still possible to rely on data from a portion of the larger study population (30). Thus, sensitivity analyzes were conducted using a multiple linear regression model, using the outcome in continuous form (BMI) (which adhered to the normal distribution after transformation by the inverse square root) and excluding low-weight individuals. Additionally, the food consumption and age group covariates were included in this model. Food consumption was obtained only in a portion of the study population (*n* = 351) from the application of the R24h, and we assessed the nutritional composition of meals of the day of each food handler through the total energy value (TEV) and macronutrients. Additionally, the sensitivity of the multinomial model was tested using the working time (independent main) in years (continuous), including age in 02 (<=40 years/> 40 years) and 5 categories (age in 5 categories: <21 years, 21–30, 31–40, 41–50, 51–59), food consumption, excluding underweight individuals and evaluating the impact on the main association. The analyzes were performed in the STATA^®^ statistical package (StataCorp LP, TX, USA), version 15.0.

### Ethical Aspects

The Ethics Committee of the University of Brasília Research approved the study (protocol 037210), and all subjects consent to participate in the study.

## Results

This study had the participation of 559 food handlers (52.6% of the total number of food handlers working in the 36 Brazilian CR). Most of the participants were female (63.2%); aged 21–40 years old (68.2%); and had a low level of education (53.3% with complete elementary school); 40.5% of food handlers had a per capita income in the household of less than half the MW (Table 1), classified as low-income (31).

**Table 1:** Descriptive analysis of socioeconomic, work, and health characteristics and anthropometric status according to body mass index (BMI) among food handlers in Brazilian community restaurants.

**Variables**	***n*** **(%)**	**Normal weight**	**Excess weight**	
			**Overweight**	**Obesity**
		***n*** **(%)**	***n*** **(%)**	***n*** **(%)**
**Gender**				
*Female*	353 (63.2)	124 (35.3)	139 (39.6)	88 (25.1)
*Male*	206 (36.8)	97 (47.3)	77 (37.6)	31 (15.1)
**Age range**				
*<40 y/o*	381 (68.2)	163 (43.0)	136 (35.9)	80 (21.1)
*>40 y/o*	178 (31.8)	58 (32.8)	80 (45.2)	39 (22.0)
**Per capita income** ^**a**^				
*>0.5 minimum wage*	320 (59.5)	134 (42.3)	125 (39.4)	58 (18.3)
*<0.5 minimum wage*	218 (40.5)	76 (34.9)	85 (38.9)	57 (26.2)
**Education**				
*High school/graduate*	261 (46.7)	121 (46.5)	92 (35.4)	47 (18.1)
*Elementary school incomplete/complete*	298 (53.3)	100 (33.8)	124 (41.9)	72 (24.3)
**Participation in a governmental program**				
*No*	281 (80.1)	176 (39.3)	184 (41.1)	88 (19.6)
*Yes*	70 (19.9)	45 (41.7)	32 (29.6)	31 (28.7)
**NCDs**
*No*	463 (82.8)	196 (42.5)	177 (38.4)	88 (19.1)
*Yes*	96 (17.2)	25 (26.3)	39 (41.1)	31 (32.6)
**Alcohol consumption**				
*No*	300 (53.8)	117 (39.3)	107 (35.9)	74 (24.8)
*Yes*	258 (46.2)	103 (40.1)	109 (42.4)	45 (17.5)
**Smoking habit**				
*No*	457 (82.1)	170 (37.4)	179 (39.3)	106 (23.3)
*Yes*	100 (17.9)	50 (50.5)	36 (36.4)	13 (13.1)
**Region of CR**				
*Midwest/Southeast/South*	320 (57.2)	115 (36.3)	129 (40.7)	73 (23.0)
*North/Northeast*	239 (42.8)	106 (44.4)	87 (36.4)	46 (19.2)
**Received any nutrition education in the company**				
*Yes*	284 (53.7)	114 (40.3)	114 (40.3)	55 (19.4)
*No*	245 (46.3)	95 (39.1)	89 (36.6)	59 (24.3)
**Years of work of food handlers**				
* < = 3 years*	484 (86.6)	200 (41.5)	179 (37.1)	103 (21.4)
*>3 years*	75 (13.4)	21 (39.8)	37 (38.8)	16 (21.4)

a *Current minimum wage at the time of the research: R$ 510.00 (175.90 USD per month)*.

Among the participants, 17.2% reported having some NCDs. Most of them do not smoke (82.1%) nor consume alcoholic beverages (53.8%). Most food handlers used to work in the foodservice sector for <3 years (86.6%), and 53.7% reported having educational activities on a healthy diet (Table 1). In addition, for a portion of the population (*n* = 351), the R24h was applied, showing that normal-weight individuals had an average daily food consumption of 2057.4 kcal (SD: 722.5, *n* = 143), and individuals with overweight and obesity had a consumption of 1829.4 kcal (SD: 649.2, *n* = 127) and 1908.8 kcal (SD: 656.4, *n* = 80), respectively. There were no statistical differences in the average daily food consumption according to BMI (*p* = 0.535). The *t*-test was applied to compare means, and no statistically significant differences were found between the food consumption of overweight and obese individuals (*p* = 0.37) nor between normal weight and obese individuals (*p* = 0.15). However, between eutrophic and overweight, a statistical difference was found (*p* = 0.00). There is a significant difference between the mean consumption in Kcal comparing individuals and their working time <36 months and > = 36 months; *p* = 0.02). Considering the working time, there was an average food consumption of 1969.8 kcal (*SD*: 702.9, *n* = 318) and 1661.5 kcal (*SD*: 412.4, *n* = 33) for individuals with 36 months or less and those with more than 36 months of work in the CR, respectively. This last analysis relating consumption and working time was not evaluated separately by gender or age.

The bivariate analysis (Table 2) was performed considering the anthropometric status as the outcome based on the BMI. Analyzing the association of variables with obesity, being male reduced the chance of obesity by 55% (OR: 0.45; CI95%: 0.28–0.73) compared to females. Individuals aged 40 years or more were 1.65 times more likely to be overweight (Odds ratio - OR: 1.65;CI95%: 1.10–2.49) than those under 40 y/o. There was no significant association between the age group and obesity (Odds ratio - OR: 1.37; CI95%: 0.84–2.23). There was no significant association between family per capita income and overweight (Odds ratio - OR: 1.20; CI95%: 0.81–1.78). There was also an association with family per capita income (OR: 1.73; CI95%: 1.09–2.75). Food handlers whose per capita income was ≤ 0.5 MW had a 73% higher chance of obesity than those with higher income.

**Table 2:** Bivariate analysis of socioeconomic, work, and health characteristics and anthropometric status according to body mass index (BMI) among food handlers in Brazilian community restaurants.

**Variables**	**Excess weight**			
	**Overweight**		**Obesity**	
	OR (CI95%)*	***P***	OR (CI95%)*	***P***
**Gender**				
*Male*	0.71 (0.48–1.04)	0.08	0.45 (0.28–0.73)	0.01
**Age range**				
*>40 y/o*	1.65 (1.10−2.49)	0.02	1.37 (0.84–2.23)	0.20
**Per capita income** ^**a**^				
*<0.5 minimum wage*	1.20 (0.81–1.78)	0.37	1.73 (1.09–2.75)	0.02
**Education**				
*Elementary school incomplete/complete*	1.63 (1.12–2.38)	0.01	1.85 (1.18–2.92)	0.01
**Participation in a governmental program**				
*Yes*	0.68 (0.41–1.11)	0.13	1.38 (0.82–2.32)	0.23
**NCDs**				
*Yes*	1.73 (1.00–2.97)	0.05	2.76 (1.54–4.95)	0.00
**Alcohol consumption**				
*Yes*	1.16 (0.80–1.69)	0.447	0.69 (0.44–1.09)	0.11
**Smoking habit**				
*Yes*	0.68 (0.42–1.10)	0.12	0.42 (0.22–0.80)	0.01
**Region of CR**				
*North/Northeast*	0.73 (0.5–1.06)	0.11	0.68 (0.43–1.07)	0.1
**Received any nutrition education in the company**				
*No*	0.94 (0.64–1.38)	0.74	1.29 (0.81–2.03)	0.28
**Years of work of food handlers**				
*>3 years*	1.96 (1.11–3.49)	0.02	1.48 (0.74–2.96)	0.27

* *OR, Odds Ratio; CI, confidence interval*.

a *Current minimum wage at the time of the research: R$ 510.00 (175.90 USD per month)*.

The educational level and NCD were associated with overweight and obesity. Concerning overweight and educational level (OR: 1.63; CI95%: 1.12–2.38), food handlers with less education (incomplete/complete elementary school) are 63% more likely to be overweight, compared with those with higher education, with an increase in the chances to 85% when evaluated for individuals with obesity (OR: 1.85; CI95%: 1.18–2.92). In the case of NCDs' presence (OR: 1.73; CI95%: 1.00–2.97), those who have some NCDs are 73% more likely to be overweight than those who do not have health problems. Individuals with NCDs had a 176% greater chance of being obese than those who did not have health problems (OR: 2.76; CI95%: 1.54–4.95). Covariables such as alcohol consumption, participation in government social programs, CR region, and nutritional education at the company showed no significant association with the outcome variable (overweight and obesity) in the bivariate analysis, but those with a *p* < 0.20 were considered for the regression model.

Table 3 shows the results of the multivariate polytomous regression model. Working in CR for 3 years or more increases the chance of being overweight by 96% compared to those who work for <3 years (OR: 1.96; CI95%: 1.11–3.49). After adjusting for the variables gender, education, income, smoking, NCDs, and the term interaction education^*^smoking, this chance remained at 88% (OR: 1.88; CI95%: 1.04–3.38). Including the variable age in the model to test the robustness of the analysis, this chance remained at 82% (OR: 1.82; CI95%: 1.01–3.27). Working more than 3 years in the foodservice sector increases the chance of being overweight by 1.80 times compared to food handlers who work for a shorter period. No significant association was found between the years of work of food handlers in the foodservice and obesity.

**Table 3:** Relationship between years of work of food handlers in the foodservice and anthropometric status in workers of community restaurants.

**Years of work as food handlers in the foodservice**	**Anthropometric status** **OR (CI 95%);** ***p*** value*	
	**Overweight**	**Obesity**
**Crude model**		
< = 3 years	Reference	Reference
>3 years	1.96 (1.11–3.49); 0.02	1.48 (0.74–2.96); 0.26
Pseudo *R*^2^	0.47%	0.47%
Adjusted model**		
< = 3 years	Reference	Reference
>3 years	1.88 (1.04–3.38); 0.03	1.25 (0.61–2.58); 0.55
Pseudo *R*^2^	4.3%	4.3%
Adjusted model***		
< = 3 years	Reference	Reference
>3 years	1.82 (1.01–3.27); 0.047	1.25 (0.60–2.58); 0.554
Pseudo *R*^2^	4.54%	4.54%

Sample size: n = 533; ^*^Multinomial Logistic Regression Model. OR, Odds Ratio; ^**^Adjusted by gender, education, per capita income, smoking, NCD, and interaction term education ^*^smoking. ^***^Adjusted by age, gender, education, per capita income, smoking, NCD, and interaction term education ^*^smoking.

The terms interaction (all covariates associated with the main exposure—working time) were tested. The best fit of the model was with the variables education and smoking and the respective interaction between them. It means that by removing the interaction between the variables, education, and smoking, the relationship between exposure—years of work of food handlers in the foodservice and the overweight outcome worsens the adjustment of the model.

Sensitivity analysis is an interesting strategy to observe the behavior of a model. Many different approaches are described, varying according to the experimental design used and how the data and results are processed. We chose to use the categorical outcome in this study since BMI is recognized as an asymmetric variable. The approach in multinominal models is usual and allows capturing the categories widely used in the scientific literature. One way to test the sensitivity of the multivariate model was to use the model with the continuous outcome and transform it to adhere to the Gaussian curve (inverse square root).

The association between working time and BMI was different for multinomial and linear regression. In linear regression, the association was negative and significant, adjusted for gender, education, per capita income, smoking, NCD, interaction term education^*^smoking, age, consumption of food, and excluding the low-weight individuals (intercept: −0.0050, *p* = 0.042). In the multinomial, the association increased among those who worked for more than 36 months and were characterized as overweight and significantly. It did not occur the same for individuals with obesity, even after the inclusion/modification of other variables. Table 4 presents four models of sensitivity developed in a subsample of the study that had its food consumption measured (reduced the sample size). The variable continuous working time and age were also included in two or five categories excluding or not individuals with low weight. In the four models, the association between working time and BMI remains.

**Table 4:** Sensitivity models performed with a subsample of the study population.

**Years of work as food handlers in the foodservice**	**Anthropometric status**		***Pseudo R*** **^2^**	**AIC**	**BIC**
	**OR (CI 95%);** p*value				
Models for sensitivity analysis*	**Overweight**	**Obesity**			
Sensitivity model 1: Time (years), kcal, age (2 categories), kept the underweight (*n* = 336)	1.24 (1.05–1.46); 0.013	1.09 (0.88–1.36); 0.42	6.17%	716.73	793.07
Sensitivity model 2: Time (years), kcal, Age (2 categories), excluding underweight individuals (*n* = 331)	1.24 (1.04–1.47); 0.014	1.10 (0.88–1.37); 0.40	6.45%	706.10	782.14
Sensitivity model 3: Time (years), kcal, Age in 5 categories, keeping underweight (*n* = 336)	1.21 (1.02–1.43); 0.03	1.07 (0.86–1.34); 0.54	6.68%	725.04	824.29
Sensitivity model 4: Time (years), kcal, Age in 5 categories, excluding underweight individuals (*n* = 331)	1.21 (1.02–1.43); 0.03	1.07 (0.86–1.34); 0.53	6.81%	715.53	814.39

*AIC, Akaike's information criterion; BIC, Bayesian information criterion. Reference: <= 3 years*.

* Variables of the main model maintained in the other models tested: gender, education, per capita income, smoking, NCD, and interaction term education ^*^smoking.

Due to the greater reliability of the results presented in the multinomial regression, especially for critical categorical outcomes such as BMI, we adopted this model mainly because the results of the linear regression present an inverse, albeit significant, relationship, which is different from the theoretical data that support the study.

## Discussion

This study is the first nationwide to focus on the association between the years of work of food handlers in the foodservice and excess weight among Brazilian food handlers from CR of all the Brazilian regions. This study is relevant considering that several complex contributors to the obesity epidemic involve work characteristics (6). This study showed a prevalence of 59.9% of excess weight (38.6% overweight and 21.3% obese) among the participants from the foodservice environment, higher than the values found for the general Brazilian population (a total of 55.4%, being 35.1% overweight and 20.3% obese) (22). Males have a smaller chance of obesity than females in our study. According to the last Budget research in Brazil (32), 30.2% of the women present obesity, while for men the percentage is lower (22.8%). When analyzing data for the population over 40 years, the percentage of overweight and obesity among females is higher. Our results are similar to studies that found a prevalence of overweight between 34.6 and 46.5% and the prevalence of obesity between 22.3 and 25.7% (9, 11, 33).

Also, our study's prevalence of excess weight is higher than the values for Canadian nationwide food services workers, which identified about 30% of excess weight among food handlers (34). In Brazil, another study (33) showed similar results to our study evaluating university restaurants' food handlers, with 56.0% of excess weight among the studied sample (*n* = 29). Other studies carried out with food handlers showed the prevalence of excess weight above 60% (10, 11). A study in the Brazilian CR from Belo Horizonte (Minas Gerais, Brazil) showed 66.7% of excess weight among the participants (*n* = 180) (31).

Our data showed a higher chance of excess weight among food handlers with years of work in the foodservice ≥3 years. Also, the bivariate analysis found an association between overweight and age >40 years. A study among US food handlers (*n* = 1,005) showed a prevalence of 23.1% of obesity (similar to our study), with a significant association with working in the food sector (6). Similar results were found among food handlers (*n* = 180) in a study with a CR in Belo Horizonte/Brazil, which also identified higher overweight chances in individuals older than 40 years (31). We found an association between overweight and obesity with a lower education level among food handlers, as found by other studies (9, 11). Also, in a study with the general Brazilian population (3), overweight and obesity had a higher prevalence among individuals with less education.

In our study, there was also an association between low-income food handlers (up to ½ MW per capita) and obesity in bivariate analysis (covariate maintained in the model after regression analysis). However, the association of the model occurred to overweight. In another study, with a small sample of CR food handlers, a lower occurrence of overweight in the highest income levels was found (34), corroborating our findings.

Our study also found an association between smoking and obesity. Dare, Mackay, and Pell discussed in their study with adults from the UK that the risk of obesity is higher among smokers, especially heavy smokers that have more chances of being obese than light smokers (35). A study with Japanese adults also concluded that obesity is associated with heavy and long-time smokers (36).

The main result of the present study was the relationship between overweight and years of work of food handlers in the CR, with a higher chance of presenting the outcome to those individuals who have worked for 3 years or more (regardless of gender, education, per capita income, smoking, and the presence of NCDs). Similar results were found in a Colombian study with 108 food handlers, in which the food handlers who worked in the foodservice sector for more than 3 years had a higher BMI compared to those who worked less time in the sector. The study concluded that being a food handler was a risk factor for being overweight (12).

Similarly, previous research showed a positive association between the years of work of food handlers in the foodservice and weight gain. Each year of extra work was significantly associated with a weight gain of 500 grams (11). In another study, 74.61% of workers gained an average of 5.6 kg since starting work at a university restaurant (33).

Obesity was also expected to be associated with longer working time, but we did not identify this in this study. This result is possibly due to the small number of workers who were obese (16) in a period longer than 36 months of work. The weight gain process is long-term, so by demonstrating the foodservice sector's association with overweight, these workers may progress to obesity over time. As weight gain is often cumulative, pre-obesity or obesity can set in if the worker remains in the food industry for a few years (37). It is worth noting that the weight gain in the foodservice sector among food handlers and the high turnover make it difficult to monitor these employees throughout their working lives. Overweight and obesity are associated with several clinical conditions such as diabetes and hypertension and social consequences such as feelings of rejection, shame, depression, and discrimination in the job market (38). Negative outcomes associated with obesity in the labor market are frequent. Excess weight may be associated with decreased quality of life, work productivity, and increased absenteeism (39, 40). Hofelmann and Blank (41), when evaluating the association between obesity and leaves, found that workers with obesity had a 2-fold greater prevalence of using long leaves.

These findings that relate a longer time (3 years or more) of exposure to work in the foodservice sector point to a phenomenon still unexplained in the researched literature, which may indicate three main hypotheses: (1) it would be an increase in muscle mass gain (an aspect that unfortunately was not investigated in this study nor in the studies that address the topic) because it is a work process of medium to an intense degree of physical activity; (2) the level of stress associated with meal production that favors weight gain since it influences on the regulation of appetite (42–45); and (3) permanent exposure to food, contributing to uninterrupted consumption (spending all day “pinching”) (16). These last two hypotheses can still result in a synergistic effect not studied in our research.

This study has the limitations of being a cross-sectional study that does not establish causal relationships. Moreover, overweight individuals' body composition was not evaluated to ensure excess body fat. The participation was voluntary, and some food handlers refused to participate, potentially leading to a systematic bias. The measures obtained may be underestimated due to the healthy worker effect, which is a type of selection bias that tends to underestimate the occurrence of health problems, since active workers would be healthier and fitter for work than those not working, due to health problems. Excess weight is on the rise in the general population and workers. This study does not present data related to physical activity.

The rigor of the sampling and methodological process, and the robustness of the statistical analysis, suggest that the research contributes to the knowledge about the health of food handlers, with the approach of an aspect related to work, in this case, the years of work in the foodservice, and the anthropometric status. Studies that evaluate workers in the foodservice sector, in general, recommend that nutritional education programs must be developed and applied to promote healthy habits and workers' health (10, 33), which can contribute to the prevention and control of excess weight among these workers. Another potential limitation of our study is the lack of categorization of alcohol consumption, not allowing us to confirm if the variation of the amount and frequency of alcohol consumption presents a different association to excess weight. There may be information bias for food consumption data (underestimated) since workers may have omitted data because they work in a place that produces meals and where the habit of “snacking” occurs but might not be reported in the search.

## Conclusion

This study was the first nationwide to identify the association of years of work of food handlers in Brazilian CR and excess weight. Data showed a significant association of almost twice the chance of being overweight among workers who have worked in restaurants for more than 3 years compared to those working for less time, with no association with obesity. The work environment is connected with factors associated with the origin and consolidation of excess weight. It should be considered essential for promoting an environment that encourages body weight maintenance. Obesity is a long process to settle in than overweight, and in the foodservice sector, high employee turnover makes it challenging to link obesity to working time. With the length of stay at work being shorter for a large part of the sample, the overweight data is more expressive.

The development and application of health assessment and promotion programs need reinforcements. The CR is considered a working environment conducive to health education activities, including their workers.

Since work-related factors may contribute to the high prevalence of excess weight, including working in a food handling environment, the government and employers should consider workplace interventions. These would guide the food handlers in avoiding high rates of excess weight and their consequences on public health. Some strategies are related to creating healthier menus for the CRs because food handlers would be encouraged to try healthy options and take home better examples for their families. As workers are low-income, they tend to eat more at work as they have less food at home. Therefore, CR should produce and serve healthier choices to their employees. Dietitians in this environment should pay more attention to their primary customers, the food handlers, since they have their meal at work with few options to change.

This study has a strength since it was carried out with a nationwide sample of workers (food handlers). The research contribution is due to the scarcity of studies related to the association of work factors and nutritional status, mainly in Brazil. As a general contribution, despite the limitations, it allows the expansion of knowledge about the health of food service workers. Furthermore, the results point to a possible influence of the work activity performed and weight gain and indicate new possibilities for studies with this professional category and variables that may complement the findings of this study. BMI can be a health indicator and a tool to provide insights about food handler's health and elucidate why some employees leave their jobs. It is worth mentioning that excess weight is an important driver of costs in the workplace associated with absenteeism, job change, and diseases. Given the findings, a reflection on health care aimed at food handlers, given that they carry out their work activities in establishments for the promotion of health and food and nutrition security, but who are not seen as part of the vulnerable population to which these services are destined.

## Data Availability Statement

The original contributions presented in the study are included in the article/supplementary material, further inquiries can be directed to the corresponding authors.

## Ethics Statement

The studies involving human participants were reviewed and approved by the Ethics Committee of the University of Brasília Research approved the study (protocol 037210), and all subjects consent to participate in the study. The patients/participants provided their written informed consent to participate in this study.

## Author Contributions

IF, RA, RRFB, and JS: conceptualization and validation. IF, RA, RRFB, JS, RZ, PC, and RBAB: methodology. IF, RA, RRFB, JC, RZ, and RBAB: formal analysis. IF, RA, RZ, and RBAB: investigation and writing—original draft preparation. IF and RA: data curation. IF, RA, RZ, HH, AR, PC, and RBAB: writing—review and editing. AR: visualization. RA: supervision. RA, RRFB, HH, AR, AA-M, and AV-M: project administration. HH, AR, AA-M, and AV-M: funding acquisition. All authors have read and agreed to the published version of the manuscript. All authors contributed to the article and approved the submitted version.

## Conflict of Interest

The authors declare that the research was conducted in the absence of any commercial or financial relationships that could be construed as a potential conflict of interest.

## Publisher's Note

All claims expressed in this article are solely those of the authors and do not necessarily represent those of their affiliated organizations, or those of the publisher, the editors and the reviewers. Any product that may be evaluated in this article, or claim that may be made by its manufacturer, is not guaranteed or endorsed by the publisher.
